# Clinical manifestations of Henoch–Schönlein purpura nephritis and IgA nephropathy: comparative analysis of data from the Japan Renal Biopsy Registry (J-RBR)

**DOI:** 10.1007/s10157-015-1177-0

**Published:** 2015-10-11

**Authors:** Hiroyuki Komatsu, Shouichi Fujimoto, Norishige Yoshikawa, Hiroshi Kitamura, Hitoshi Sugiyama, Hitoshi Yokoyama

**Affiliations:** First Department of Internal Medicine, University of Miyazaki Hospital, 5200 Kihara, Kiyotake, Miyazaki, 889-1692 Japan; Department of Hemovascular Medicine and Artificial Organs, Faculty of Medicine, University of Miyazaki, Miyazaki, Japan; Center for Clinical Research and Development, National Center for Child Health and Development, Tokyo, Japan; Department of Pathology, Clinical Research Center, National Hospital Organization Chiba East National Hospital, Chiba, Japan; Department of Medicine and Clinical Science, Okayama University Graduate School of Medicine, Dentistry, and Pharmaceutical Science, Okayama, Japan; Division of Nephrology, Kanazawa Medical University School of Medicine, Ishikawa, Japan

**Keywords:** Henoch–Schönlein purpura nephritis, IgA nephropathy, Renal biopsy, Registry, Glomerulonephritis, Age distribution

## Abstract

**Background:**

The clinical presentation of Henoch–Schönlein purpura nephritis (HSPN) has not been thoroughly investigated among patients of different ages. We therefore compared the features of HSPN and IgA nephropathy (IgAN) based on data from the Japan Renal Biopsy Registry (J-RBR).

**Methods:**

This cross-sectional study analyzed data from patients who were registered in the J-RBR between 2007 and 2012. Clinico-pathological findings at diagnosis were compared among children (aged ≤18 years), adult (aged 19–64 years) and elderly (aged ≥65 years) patients with HSPN (*n* = 513) and IgAN (*n* = 5679).

**Results:**

The age at diagnosis considerably differed between HSPN and IgAN; HSPN peaked at 1–19 and at 60–69 years, whereas IgAN peaked at 30–39 years. The clinical features were significantly more severe for HSPN than IgAN, especially proteinuria (children, 1.28 vs. 0.57; adult, 1.95 vs. 1.05; elderly patients, 2.71 vs. 1.64 g/day), and low albumin levels (children, 3.72 vs. 4.13; adults, 3.62 vs. 3.99; elderly patients, 3.07 vs. 3.57 g/dL). The rate (%) of histologically classified endocapillary proliferative or crescentic glomerulonephritis was higher in patients with HSPN than with IgAN. Multiple regression analysis revealed that low albumin level and high BP were independent factors associated with decreased estimated glomerular filtration rates in adult and elderly patients with HSPN.

**Conclusions:**

Age at HSPN diagnosis was bimodally distributed, and the clinical features of HSPN were more severe than those of IgAN across all age groups.

## Introduction

Immunoglobulin A nephropathy (IgAN) is one of the most prevalent types of glomerulonephritis, especially in East Asia, Europe, and North America [1–3]. In contrast, Henoch–Schönlein purpura (HSP) is a type of vasculitis that frequently arises in children. About 30–60 % of patients with HSP also develop nephritis (HSPN) with urinary abnormalities and/or renal impairment [4].

Whether or not IgAN and HSPN are related has been controversial [5], but several progressive studies have shown that aberrantly glycosylated IgA1 is responsible for the onset of both diseases [6–9]. Consequently, the eponym “HSP” was replaced with “IgA vasculitis” in the revised International Chapel Hill Consensus Conference nomenclature of vasculitis [10, 11].

Renal function and histological damage at diagnosis, hypertension and heavy proteinuria at diagnosis and during disease progression are established prognostic factors for both HSPN and IgAN [3, 12–17]. Some studies have compared the clinical and pathological findings between IgAN and HSPN [18, 19]. However, these studies included relatively few participants with age restrictions, and thus the clinical presentation of HSPN has not been thoroughly assessed in an adequate sample of patients at different ages.

A nationwide, web-based, prospective registry of renal biopsies (Japan Renal Biopsy Registry; J-RBR) was established during 2007 in Japan, and data from about 20,000 patients have been registered [20, 21]. Hence, the purpose of this study is to clarify the differences and relationship of clinico-pathological findings between IgAN and HSPN by using this nationwide and large database.

## Materials and methods

### Outline of J-RBR system and selection of patients

The J-RBR was established by the Committee for the Standardization of Renal Pathological Diagnosis and the Working Group for the Renal Biopsy Database of the Japanese Society of Nephrology in 2007. Patients’ data were registered on the J-RBR website using the Internet Data and Information Center for Medical Research (INDICE) system of the University Hospital Medical Information System (UMIN). The J-RBR is registered under the Clinical Trial Registry of UMIN (Registration Number, UMIN000000618), and the Ethics Review Board of the Japanese Society of Nephrology approved the present study in accordance with the Declaration of Helsinki.

The main registration system comprised basic information of the patients, date and number of renal biopsy, pathological information based on pathogenesis and histopathology, urinary and blood findings, and coexisting of hypertension and diabetes. Among 18,967 patients with biopsy-proven disease who were registered in this system between July 2007 and December 2012, we selected 513 with HSPN and 5679 with IgAN, which were registered in IgAN or HSPN as the pathogenesis. The two groups of patients were classified as children, adults, and elderly according to ages ≤18, 19–64. and ≥65 years, respectively.

### Clinical and pathological diagnoses

Primary glomerular diseases were mainly clinically diagnosed as chronic nephritic syndrome, acute nephritic syndrome, recurrent or persistent hematuria, rapidly progressive nephritic syndrome and nephrotic syndrome, according to the modified classification of World Health Organization [20, 21]. Secondary and tubulo-interstitial diseases were categorized as renal disorders with collagen disease or vasculitis, renal disease with metabolic syndrome, hypertensive nephropathy, acute kidney injury, drug-induced nephropathy, thrombotic microangiopathy, and others (including acute/chronic interstitial injury, acute tubular necrosis).

The J-RBR requires classification based on pathogenesis and histopathology. The histopathology of HSPN and IgAN, which comprised the pathogenesis of all our patients, was evaluated as mesangial proliferative glomerulonephritis, endocapillary proliferative glomerulonephritis, minor glomerular abnormalities, focal segmental glomerulosclerosis, membranous nephropathy, membranoproliferative glomerulonephritis (types I and III), dense deposit disease (type II), crescentic and necrotizing glomerulonephritis, sclerotic glomerulonephritis, nephrosclerosis, acute/chronic interstitial nephritis, renal transplantation, and others.

### Evaluation of other clinical findings

The registered basic information (age, sex, height, and weight), as well as urinary findings (urinalysis, daily proteinuria), blood findings [serum creatinine (sCr), total protein, serum albumin, total cholesterol], and blood pressure (BP) were assessed in the present study. Estimated glomerular filtration rates (eGFR) were calculated using the modified equation for Japanese [22]. Information about prescribed anti-hypertensive agents, the presence of diabetes mellitus and HbA1c that was arbitrarily registered was insufficient and could not be assessed.

### Statistical analysis

Continuous variables, except age, are presented as mean ± standard deviation (SD). Age is expressed as median and interquartile range. Clinical parameters were compared between the groups with HSPN and IgAN using the unpaired *t* test for normally distributed continuous variables or the Mann–Whitney *U* test for non-normally distributed continuous variables. Clinical parameters were compared among the three age groups using a single-factor analysis of variance (ANOVA) for normally distributed continuous variables or the Kruskal–Wallis test for non-normally distributed continuous variables. The normality of the variances for each continuous variable was analyzed by the Levene test. Differences in proportions were evaluated using the Chi-square independent test or Fisher’s exact test, depending on the number of categories. Independent factors affecting renal function at diagnosis were evaluated using stepwise multiple regression analyses. Quantitative variables such as body mass index (BMI) calculated by height and weight, proteinuria, systolic BP, serum albumin, and serum total cholesterol were selected as independent variables in the analyses. Age, sex, and the value of sCr were excluded, because these variables were used in the equation of eGFR. All data were statistically analyzed using IBM SPSS Advance Statistical version 22.0 and *p* < 0.05 was considered to indicate a significant difference.

## Results

### Age distribution differs between HSPN and IgAN

**Fig. 1:**
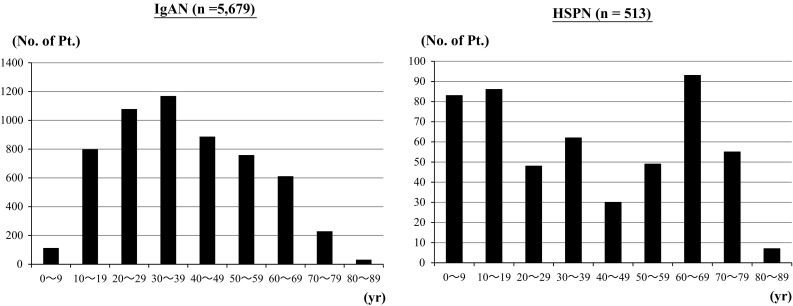
Age distribution of IgA nephropathy (*n* = 5679) and Henoch–Schönlein purpura nephritis (*n* = 513). Each *histogram* shows 10-year intervals. Frequency of HSPN diagnosis is bimodal with peaks at 1–19 and 60–69 years; frequency of IgAN diagnosis peaks at 30–39 years

Figure 1 shows the age distribution of the patients with HSPN and IgAN. The median ages of the HSPN and IgAN groups were almost equivalent (36 years in HSPN and 37 years in IgAN) but the distribution differed between the two groups, as HSPN peaked at 1–19 and at 60–69 years, whereas IgAN peaked at 30–39 years. There were no remarkable differences of the age distribution by gender in both the diseases.

### Comparison of clinico-pathological diagnoses and parameters between HSPN and IgAN

Table 1 compares the clinico-pathological diagnoses and clinical findings between the two diseases. The ratio of chronic nephritic syndrome was significantly higher in patients with IgAN than in those with HSPN (88.5 vs. 61.6 %), whereas those of rapidly progressive nephritic syndrome and nephrotic syndrome were significantly higher in patients with HSPN than in those with IgAN (4.5 vs. 1.4 % and 10.5 vs. 3.0 %, respectively). Mesangial proliferative glomerulonephritis was pathologically evident in 90 % of patients with IgAN. The ratios of endocapillary proliferative glomerulonephritis or crescentic and necrotizing glomerulonephritis were significantly higher, and the clinical findings were far worse in the group with HSPN than in that with IgAN [proteinuria, 1.93 ± 2.53 vs. 1.05 ± 1.40 g/day; ratio of overt hematuria (sediment RBC >30/HPF), 50.3 vs. 36.8 %; serum albumin levels, 3.55 ± 0.75 vs. 3.97 ± 0.56 g/dL). The ratio of patients with more advanced chronic kidney disease stages (grade 3–5) was also higher in HSPN, as compared to IgAN.

**Table 1:** Comparison of clinico-pathological diagnoses and parameters between HSPN and IgAN (*n* = 6192)

	IgAN (*n* = 5679)	HSPN (*n* = 513)	*p*
Age [median, (interquartile range)]	37 (24–52)	36 (14–62)	0.41
Gender (male/female)	2887/2792	244/269	0.16
Clinical diagnosis			
Chronic nephritic syndrome	5028 (88.5 %)	316 (61.6 %)	<0.001*
Acute nephritic syndrome	74 (1.3 %)	29 (5.7 %)	<0.001*
Recurrent or persistent hematuria	269 (4.7 %)	17 (3.3 %)	0.14
Rapidly progressive nephritic syndrome	79 (1.4 %)	23 (4.5 %)	<0.001*
Nephrotic syndrome	169 (3.0 %)	54 (10.5 %)	<0.001*
Renal disorder with collagen disease or vasculitis	10 (0.2 %)	63 (12.3 %)	<0.001*
Hypertensive nephropathy	5 (0.1 %)	0 (0.0 %)	0.50
Acute renal failure	8 (0.1 %)	0 (0.0 %)	0.40
Renal transplantation	7 (0.1 %)	0 (0.0 %)	0.43
Others (including metabolic and drug-induced)	30 (0.5 %)	11 (2.1 %)	<0.001*
Pathological diagnosis			
Mesangial proliferative GN	5259 (92.6 %)	397 (77.4 %)	<0.001*
Endocapillary proliferative GN	49 (0.9 %)	33 (6.4 %)	<0.001*
Minor glomerular abnormality	115 (2.0 %)	22 (4.3 %)	0.001*
Focal segmental glomerulosclerosis	39 (0.7 %)	1 (0.2 %)	0.18
Membranous nephropathy	14 (0.2 %)	1 (0.2 %)	0.82
Membranoproliferative GN (type I and III)	21 (0.4 %)	5 (1.0 %)	0.04*
Dense deposit disease	3 (0.1 %)	0 (0.0 %)	0.60
Crescentic and necrotizing GN	46 (0.8 %)	34 (6.6 %)	<0.001*
Sclerosing GN	28 (0.5 %)	0 (0.0 %)	0.11
Nephrosclerosis	25 (0.4 %)	0 (0.0 %)	0.13
Acute/chronic interstitial nephritis	6 (0.1 %)	1 (0.2 %)	0.56
Renal transplantation	3 (0.1 %)	0 (0.0 %)	0.60
Others	71 (1.3 %)	19 (3.7 %)	<0.001*
Clinical findings			
Body mass index	22.5 ± 3.92	21.8 ± 4.95	0.004*
Systolic BP (mmHg)	123.3 ± 18.0	122.6 ± 19.8	0.48
Diastolic BP (mmHg)	74.1 ± 13.1	71.8 ± 13.0	<0.001*
Pt. with hypertension at diagnosis	1982 (34.9 %)	186 (36.3 %)	0.43
Sediment RBC (>30/HPF, %)	2090 (36.8 %)	258 (50.3 %)	<0.001*
Proteinuria (g/day)	1.05 ± 1.40	1.93 ± 2.53	<0.001*
Proteinuria (g/gCr)	1.44 ± 1.93	3.04 ± 3.39	<0.001*
Serum creatinine (mg/dL)	0.98 ± 0.72	0.91 ± 0.88	0.12
Estimated GFR (>20 years old, *n* = 5098)	69.0 ± 26.3	66.2 ± 30.1	0.10
CKD stage (>20 years old, *n* = 5098)			0.008*
Grade 1 (eGFR >90/mL/min/1.73 m^2^)	1028 (21.6 %)	72 (21.0 %)	
Grade 2 (eGFR 60–89/mL/min/1.73 m^2^)	1945 (40.9 %)	115 (33.5 %)	
Grade 3 (eGFR 30–59/mL/min/1.73 m^2^)	1434 (30.2 %)	117 (34.1 %)	
Grade 4 (eGFR 15–29/mL/min/1.73 m^2^)	270 (5.7 %)	28 (8.2 %)	
Grade 5 (eGFR <15/mL/min/1.73 m^2^)	78 (1.6 %)	11 (3.2 %)	
Serum albumin (g/dL)	3.97 ± 0.56	3.55 ± 0.75	<0.001*
Serum total cholesterol (mg/dL)	199.5 ± 46.5	212.4 ± 60.4	<0.001*

*GN* glomerulonephritis, *CKD* chronic kidney disease

* *p* < 0.05 by un-paired *t* test or Chi-square test or Fisher’s exact test

### Comparison of clinico-pathological parameters of HSPN and IgAN according to age

Table 2 compares the clinico-pathological findings among children, adult, and elderly patients with HSPN and IgAN. As for HSPN, the ratios of rapidly progressive nephritic syndrome and nephrotic syndrome (0.6 vs. 3.4 vs. 13.5 % and 5.1 vs. 10 vs. 18.8 %, respectively), and of histopathological endocapillary proliferative glomerulonephritis and crescentic/necrotizing glomerulonephritis (4.5 vs. 5.0 vs. 13.5 % and 1.9 vs. 7.3 vs. 12.5 %, respectively) were significantly higher in elderly patients than in adult and children. The number of patients with hypertension, large amounts of proteinuria, overt hematuria, reduced renal function, and hypoalbuminemia distinctly differed among the age groups, and disease severity was worse in elderly patients with HSPN.

**Table 2:** Comparison of clinico-pathological diagnoses and parameters among age group in HSPN and IgAN

	IgAN (*n* = 5679)				HSPN (*n* = 513)			
	Child (*n* = 803)	Adult (*n* = 4379)	Elderly (*n* = 497)	p*	Child (*n* = 158)	Adult (*n* = 259)	Elderly (*n* = 96)	p*
Age [median, (interquartile range)]	15 (12–17)	38 (29–50)	70 (67–74)	<0.001*	9 (6–13)	43 (30–59)	72 (68–76)	<0.001*
Gender (male/female)	454/349	2115/2264	318/179	<0.001**	76/82	119/140	49/47	0.69
Clinical diagnosis								
Chronic nephritic syndrome	732 (91.2 %)	3935(89.9 %)	385 (77.5 %)	<0.001**	130 (82.3 %)	151 (58.3 %)	35 (36.5 %)	<0.001**
Acute nephritic syndrome	13 (1.6 %)	29 (0.7 %)	9 (1.8 %)	0.002**	7 (4.4 %)	18 (6.9 %)	4 (4.2 %)	0.44
Recurrent or persistent hematuria	45 (5.6 %)	206 (4.7 %)	18 (3.6 %)	0.26	4 (2.5 %)	8 (3.1 %)	5 (5.2 %)	0.49
Rapidly progressive nephritic syndrome	2 (0.2 %)	52 (1.2 %)	25 (5.0 %)	<0.001**	1 (0.6 %)	9 (3.4 %)	13 (13.5 %)	<0.001**
Nephrotic syndrome	9 (1.1 %)	111 (2.5 %)	49 (9.9 %)	<0.001**	8 (5.1 %)	28 (10.8 %)	18 (18.8 %)	0.002**
Renal disorder with collagen disease or vasculitis	0 (0.0 %)	10 (0.2 %)	0 (0.0 %)	0.23	7 (4.4 %)	38 (14.7 %)	18 (18.8 %)	<0.001**
Pathological diagnosis								
Mesangial proliferative GN	748 (93.2 %)	4070(92.9 %)	441 (88.7 %)	0.003**	129 (81.6 %)	206 (79.5 %)	62 (64.6 %)	0.003**
Endocapillary proliferative GN	6 (0.7 %)	40 (0.9 %)	3 (0.6 %)	0.72	7 (4.5 %)	13 (5.0 %)	13 (13.5 %)	0.007**
Minor glomerular abnormality	34 (4.2 %)	72 (1.6 %)	9 (1.8 %)	<0.001**	15 (9.5 %)	6 (2.3 %)	1 (1.0 %)	<0.001**
Focal segmental glomerulosclerosis	1 (0.1 %)	34 (0.8 %)	4 (0.8 %)	0.11	0 (0.0 %)	1 (0.4 %)	0 (0.0 %)	0.61
Membranous nephropathy	1 (0.1 %)	9 (0.2 %)	4 (0.8 %)	0.03**	0 (0.0 %)	1 (0.4 %)	0 (0.0 %)	0.61
Membranoproliferative GN (type I and III)	0 (0.0 %)	17 (0.4 %)	4 (0.8 %)	0.06	1 (0.6 %)	3 (1.2 %)	1 (1.0 %)	0.87
Crescentic and necrotizing GN	7 (0.9 %)	29 (0.7 %)	10 (2.0 %)	0.006**	3 (1.9 %)	19 (7.3 %)	12 (12.5 %)	0.004**
Sclerosing GN	1 (0.1 %)	22 (0.5 %)	5 (1.0 %)	0.09	0 (0.0 %)	1 (0.4 %)	0 (0.0 %)	0.61
Clinical findings								
Body mass index	19.9 ± 3.83	22.9 ± 3.82	23.4 ± 3.31	<0.001*	18.0 ± 3.89	23.5 ± 4.59	23.5 ± 3.91	<0.001*
Systolic BP (mmHg)	110.5 ± 12.0	123.9 ± 17.5	136.6 ± 18.4	<0.001*	106.4 ± 11.1	125.6 ± 17.5	138.5 ± 19.2	<0.001*
Diastolic BP (mmHg)	64.0 ± 9.48	75.4 ± 13.0	76.8 ± 11.2	<0.001*	62.7 ± 9.97	75.1 ± 12.0	76.6 ± 12.9	<0.001*
Pt. with hypertension at diagnosis	52 (6.5 %)	1619 (37.0 %)	311 (62.6 %)	<0.001**	14 (8.9 %)	107 (41.3 %)	65 (67.7 %)	<0.001**
Sediment RBC (>30/HPF, %)	411 (51.2 %)	1495 (34.1 %)	184 (37.0 %)	<0.001**	73 (46.2 %)	125 (48.3 %)	60 (62.5 %)	0.03**
Proteinuria (g/day)	0.57 ± 1.05	1.05 ± 1.36	1.64 ± 1.86	<0.001*	1.28 ± 2.04	1.95 ± 2.54	2.71 ± 2.88	0.001*
Proteinuria (g/gCr)	0.93 ± 1.51	1.38 ± 1.72	2.74 ± 3.27	<0.001*	3.23 ± 4.03	2.53 ± 2.84	4.08 ± 3.64	<0.001*
Serum creatinine (mg/dL)	0.63 ± 0.41	0.99 ± 0.69	1.39 ± 1.05	<0.001*	0.49 ± 0.72	0.91 ± 0.45	1.62 ± 1.64	<0.001*
Estimated GFR (mL/min/1.73 m^2^)	–	71.6 ± 25.7	46.8 ± 19.7	<0.001***	–	74.2 ± 28.6	45.4 ± 24.3	<0.001***
Serum albumin (g/dL)	4.13 ± 0.54	3.99 ± 0.52	3.57 ± 0.67	<0.001*	3.72 ± 0.76	3.62 ± 0.74	3.07 ± 0.59	<0.001*
Serum total cholesterol (mg/dL)	179.6 ± 42.7	202.5 ± 45.7	206.2 ± 50.8	<0.001*	208.1 ± 63.1	215.1 ± 58.4	212.3 ± 61.2	0.53

*GN* glomerulonephritis

* *p* < 0.05 by one way ANOVA or Kruskal–Wallis test

** *p* < 0.05 by Chi-square test

*** *p* < 0.05 by unpaired *t* test

The clinical findings such as hypertension, proteinuria, and renal function were similarly worse in elderly patients with IgAN. In contrast, the ratio of endocapillary proliferative glomerulonephritis (0.7 vs. 0.9 vs. 0.6 %) in IgAN did not differ among the age groups (Table 2).

### Comparison of clinico-pathological parameters between HSPN and IgAN according to age

In all age groups, the patients with HSPN had more proteinuria (children, 1.28 vs. 0.57 g/day; adult, 1.95 vs. 1.05 g/day; elderly patients, 2.71 vs. 1.64 g/day; *p* ≤ 0.001), and low albumin level (children, 3.72 vs. 4.13 g/dL; adults, 3.62 vs. 3.99 g/dL; elderly patients, 3.07 vs. 3.57 g/dL; *p* ≤ 0.001) than those with IgAN. Moreover, the ratios of histopathological endocapillary proliferative glomerulonephritis (13.5 vs. 0.6 %, *p* ≤ 0.001) and crescentic glomerulonephritis (12.5 vs. 2.0 %, *p* ≤ 0.001) were distinctly higher in elderly patients with HSPN than in those with IgAN.

### Effect of clinico-pathological factors on renal function at the time of diagnosis in adult and elderly patients with HSPN and IgAN

The effects of clinico-pathological factors on renal function at the time of diagnosis were evaluated using multiple regression analysis. The model included important risk factors for IgAN progression as imperative independent variables. Low albumin level and higher value for systolic BP values at diagnosis were significant factors for a decline in renal function in adult and elderly patients with HSPN (*R*^2^ = 0.190; *F* = 10.54, *p* < 0.05; Table 3a). Increased proteinuria, lower albumin level, higher value for BMI, and systolic BP were significant factors for a decline in renal function in adult and elderly patients with IgAN (*R*^2^ = 0.236; *F* = 180.7; *p* < 0.05; Table 3b).

**Table 3:** Effect of clinico-pathological factors on renal function at the time of diagnosis in adult and elderly patients with IgAN and HSPN

Parameters at diagnosis	Standard *β*	*t*	*p* value
(a) HSPN			
Serum albumin (g/dL)	0.274	3.428	0.001*
Systolic BP (mmHg)	−0.214	−2.912	0.004*
*R* ^2^ = 0.190, *F* value = 10.54 (*p* < 0.001*)			
(b) IgAN			
Serum albumin (g/dL)	0.215	10.82	<0.001*
Systolic BP (mmHg)	−0.303	−17.17	<0.001*
Proteinuria (g/day)	−0.106	−5.081	<0.001*
Body mass index	−0.072	−4.190	<0.001*
*R* ^2^ = 0.236, *F* value = 180.7 (*p* < 0.001*)			

* Statistically significant by multiple regression analysis (stepwise method)

## Discussion

The present study of the J-RBR revealed that the distribution of age at HSPN diagnosis was bimodal with peaks at 1–19 and 60–69 years, whereas that at IgAN diagnosis peaked during the fourth decade. Patients with HSPN had a higher frequency of nephrotic syndrome and rapidly progressive nephritic syndrome at onset, histologically confirmed endocapillary proliferation and crescentic glomerulonephritis, and clinical hypertension, heavy proteinuria and hypoalbuminemia than those with IgAN. Hypoalbuminemia and higher BP level had significant relevance to the decline of renal function at diagnosis in both diseases.

A Japanese nationwide survey between 1985 and 1993 and a review published in the USA during 2002 showed that the onset of IgAN occurs during the second and third decades of life [23, 24]. The present study confirmed these findings. By contrast, the age distribution of HSPN has not been investigated in detail since the onset of HSP itself is usually during childhood. Coppo et al. reported that the mean age of 57 children and 95 adults with HSPN was 27.5 (3–72) years [12] and Pillebout et al. found that the mean age of 250 adult patients with HSPN was 50 (15–86) years [15]. However, these studies did not include information about the age distribution. The novel findings of the present study are that the frequency of HSPN peaked twice and the clinico-pathological findings were more severe at the peak age of 60–69 years than at the other peak age of 1–18 years.

Whether or not disease severity at diagnosis and prognosis differs between HSPN and IgAN remains controversial. Although the present study found that almost all clinical findings at diagnosis were more severe in all age groups of patients with HSPN than IgAN, we could not investigate the relationship between the severity at diagnosis and prognosis. Oh et al. indicated that clinical outcomes did not vary between adult onset HSPN (*n* = 89) and IgAN (*n* = 178) after clinical severity at presentation was matched [19]. Conversely, Rio et al. found more severe renal involvement in patients with IgAN (*n* = 61) than with HSPN (*n* = 142) after a 10-year follow-up [25]. Further studies are needed to elucidate relationship between the severity at diagnosis and prognosis.

The histological features of HSPN and IgAN are considered difficult to distinguish without other extra-renal findings, because light microscopic finding shows similar mesangial proliferation and IgA deposition by the immune-fluorescence staining. Therefore, debate about whether or not IgAN and HSPN are related has persisted for several years [4, 5]. Recent findings have indicated that aberrantly glycosylated IgA1 is involved in the onset of both diseases [9, 26]. Novak et al. indicated that circulating IgA1 contains galactose-deficient O-linked glycans in patients with HSPN and in those with IgAN [7]. Kiryluk also found that elevated serum levels of galactose-deficient IgA1 are inherited by pediatric patients with HSPN and IgAN [8]. Moreover, the expression of mRNAs encoding toll-like receptors that play a key role against extrinsic antigens is increased in both diseases [27]. Thus, similar factors contribute to the onset of both diseases, although they are quite different in terms of extra-renal presentation such as gastrointestinal involvement, arthritis and purpuric skin lesions. Furthermore, the present study uncovered differences in the clinical diagnosis and clinico-pathological severity of renal findings between the two diseases. These might be caused by the nature of individual immune responses to circulating immune-complexes of aberrantly glycosylated IgA1. Some other factors might also be involved in elderly patients with HSPN. However, we were unable to investigate this notion. Further studies are needed, because understanding the reasons for the differences might facilitate the development of new disease-specific therapies.

Several studies have indicated that elderly individuals with IgAN are affected not only by continuous immune activity, but also by increases in atherosclerotic changes caused by hypertension, as well as disordered lipid and glucose metabolism, and these factors synergistically affect renal involvement [28, 29]. The present study supports these findings since elderly patients with either IgAN or HSPN had more severe proteinuria and higher BP than adults and children with the same respective diseases. On the other hand, the clinico-pathological findings were more severe in elderly patients with HSPN than with IgAN. This result might suggest that the severity of HSPN in elderly patients cannot explain the known atherosclerotic changes.

The difference between the daily proteinuria (g/day) and urinary protein/creatinine ratio (g/gCr) was evident, especially in elderly and child patients with IgAN and HSPN in this study. The small effect of some missing data (10–15 % of the total) for urinary protein/creatinine ratio could not be denied, although this study had relatively large subjects. Instead, this discrepancy might have more important implication for the assessment of proteinuria in elderly and child. Yokoyama et al. previously suggested that proteinuria was overestimated by the urinary protein/creatinine ratio in the elderly because of the decreased expression of urinary creatinine brought about by the reduction of muscle mass that occurs during aging [30]. This theory might be also applicable to child since muscle mass of them are less than adults.

This study was feasible due to having access to a large database. The J-RBR was the first nationwide, prospective registry of renal biopsies and it was established in 2007; since then, about 5000 patients from 130 institutions in Japan have been registered every year [20]. The registry contributes to not only the standardization of histological diagnosis and classification, but also to nationwide epidemiological studies of conditions such as nephrotic syndrome and glomerulonephritis. In fact, a new perception of the frequency of renal diseases and the outcomes of nephrotic syndrome in elderly patients has emerged based on this system [30, 31]. We also uncovered some useful information about different age groups with HSPN and IgAN using registry data. The formulation and application of the nationwide registry will become even more valuable in the future.

This study has some limitations. Firstly, the registry data might be somewhat inaccurate. IgAN has been accompanied with the other primary glomerular diseases such as minor glomerular abnormality and focal segmental glomerulosclerosis in this study. In fact, there are some difficult cases to distinguish IgAN and other primary glomerular diseases only with pathological findings, because the pathological definition of IgAN is only mesangial proliferation with IgA deposition. Each individual registrant rather than a centralized individual was responsible for the diagnosis of HSPN. Second, the number of items on the registration form was so definitive that we were unable to evaluate the histological findings based on the Oxford classification of IgAN and the International Society of Kidney Disease classification of HSPN. Third, the difference of indication of renal biopsy among child, adult, and elderly should be considered in comparing the clinico-pathological findings at diagnosis. Finally, this cross-sectional study had no reference to the renal outcomes of any of the patients. However, the baseline data of this study have a potential to develop further studies such as a longitudinal cohort study. From this perspective, a large longitudinal cohort study should be planned to clarify and compare actual outcomes between the two diseases.

In conclusion, the clinico-pathological findings were more severe among patients with HSPN than with IgAN across three age groups. The frequency of HSPN diagnosis peaked once in childhood (<18 years of age) and again between the ages of 60 and 69 years, and the clinico-pathological findings were more severe in elderly patients than in children with HSPN. The clinico-pathological characteristics and actual renal outcomes of elderly patients with HSPN require further investigation.
